# The Brain Is Faster than the Hand in Split-Second Intentions to Respond to an Impending Hazard: A Simulation of Neuroadaptive Automation to Speed Recovery to Perturbation in Flight Attitude

**DOI:** 10.3389/fnhum.2016.00187

**Published:** 2016-04-27

**Authors:** Daniel E. Callan, Cengiz Terzibas, Daniel B. Cassel, Masa-aki Sato, Raja Parasuraman

**Affiliations:** ^1^Center for Information and Neural Networks, National Institute of Information and Communications Technology, Osaka UniversityOsaka, Japan; ^2^Multisensory Cognition and Computation Laboratory, Universal Communication Research Institute, National Institute of Information and Communications TechnologyKyoto, Japan; ^3^Locomobi Inc.Toronto, ON, Canada; ^4^Neural Information Analysis Laboratories, Advanced Telecommunications Research InstituteKyoto, Japan; ^5^Center of Excellence in Neuroergonomics, Technology, and Cognition, George Mason UniversityFairfax, VA, USA

**Keywords:** neuroadaptive automation, brain computer interface, brain machine interface, neuroergonomics, decoding, independent component analysis, MEG, aviation

## Abstract

The goal of this research is to test the potential for neuroadaptive automation to improve response speed to a hazardous event by using a brain-computer interface (BCI) to decode perceptual-motor intention. Seven participants underwent four experimental sessions while measuring brain activity with magnetoencephalograpy. The first three sessions were of a simple constrained task in which the participant was to pull back on the control stick to recover from a perturbation in attitude in one condition and to passively observe the perturbation in the other condition. The fourth session consisted of having to recover from a perturbation in attitude while piloting the plane through the Grand Canyon constantly maneuvering to track over the river below. Independent component analysis was used on the first two sessions to extract artifacts and find an event related component associated with the onset of the perturbation. These two sessions were used to train a decoder to classify trials in which the participant recovered from the perturbation (motor intention) vs. just passively viewing the perturbation. The BCI-decoder was tested on the third session of the same simple task and found to be able to significantly distinguish motor intention trials from passive viewing trials (mean = 69.8%). The same BCI-decoder was then used to test the fourth session on the complex task. The BCI-decoder significantly classified perturbation from no perturbation trials (73.3%) with a significant time savings of 72.3 ms (Original response time of 425.0–352.7 ms for BCI-decoder). The BCI-decoder model of the best subject was shown to generalize for both performance and time savings to the other subjects. The results of our off-line open loop simulation demonstrate that BCI based neuroadaptive automation has the potential to decode motor intention faster than manual control in response to a hazardous perturbation in flight attitude while ignoring ongoing motor and visual induced activity related to piloting the airplane.

## Introduction

Safe and effective performance in many occupational settings is critically dependent on people making timely and correct split-second decisions to avoid an impending hazard. Consider a speeding driver having to swerve to avoid hitting a child running unexpectedly onto the roadway; a nurse having to administer defibrillation to a patient having sudden cardiac arrest; or a pilot having to execute a rapid maneuver to recover from a stall or other abrupt perturbation during high-speed flight. Although some drivers, nurses, and pilots may be sufficiently skilled to make quick decisions and avoid mishaps in these situations, there are many conditions—fatigue, stress, mind wandering, task overload, to name a few—that can degrade human performance so that a correct and timely response is not possible and an accident may result.

One approach to this problem is to enhance human performance in such time-critical situations by decoding a person's neural activity associated with the intention to act. Once intention has been detected, one could provide appropriate feedback to the human operator or trigger computer aiding. Brain activity precedes motor action, so if neural signals associated with the intention to act could be successfully decoded in real time, one could use the decoded output to aid the human user. Using computer technology to augment human performance based on an assessment of human operator cognitive states is termed adaptive automation (Parasuraman et al., 1992; Scerbo, 2008; Parasuraman and Galster, 2013). *Neuroadaptive* automation is when neural signals are used to assess operator state, an approach that has been successful in mitigating human performance decrements in a variety of cognitive tasks (Byrne and Parasuraman, 1996; Prinzel et al., 2000; Scerbo et al., 2003; Wilson and Russell, 2007; Ting et al., 2010; Durantin et al., 2015; Gateau et al., 2015). Such an approach is consistent with the field of passive Brain Computer Interfaces (BCI), also referred to as Brain Machine Interfaces (BMI), in which user neural states are monitored in order to enhance human interaction with external devices (Blankertz et al., 2010; Lotte et al., 2013).

There is extensive research on the use of BCIs to support partially or fully disabled persons to control devise such as computers and prosthetic limbs (Reiner, 2008), and a smaller but growing literature on their use for healthy individuals so as to enhance human-system interaction (Zander and Kothe, 2011; Lotte et al., 2013). Comparatively little work has been conducted comparing the effects of neuroadaptive automation or passive BCIs to human performance in time-critical (split-second) decision-making situations [For related research see the studies by Haufe et al. (2011, 2014) and Kim et al. (2015) concerned with detection of braking intention by EEG]. In particular, when a critical event has to be detected and responded to quickly, can one decode the associated neural states of the human operator to achieve a faster response than the operator's manual action? We can rephrase the question as follows: given that the brain is faster than the hand (or foot or other effector), can one solve the problem that human manual actions are sometimes too sluggish to avoid a mishap when very little time is available by using the decoded brain activity to respond to a critical hazardous event?

We addressed this issue in the present study by examining whether neural signals could be decoded to enhance human performance in a time-critical decision-making task. We chose a decision-making situation that is encountered in aviation tasks: responding quickly to an in-flight perturbation, such as turbulence, micro-bursts, severe windshear, structural damage (e.g., from trim tab failure, bird strike, etc.). While such perturbations can occur in many types of flight, they can be a major contributor to mishaps in military aviation, given the greater exposure to risky situations requiring split-second decision-making, such as low-level flight over terrain, or high G-force maneuvers (Knapp and Johnson, 1996; Moroze and Snow, 1999; Nakagawa et al., 2007). When flying at high speed and very close to terrain, a savings of even a few milliseconds in responding to a perturbation can represent the difference between life and death (Haber and Haber, 2003).

Decoding neural states corresponding to cognitive states has been the object of considerable attention in the neuroimaging literature. A major approach to the problem has been to apply pattern-classification algorithms to multi-voxel functional MRI data in order to decode information representation in a participant's brain (Kamitani and Tong, 2005, 2006; Norman et al., 2006; Nishimoto et al., 2011; Poldrack, 2011; Shibata et al., 2012; Callan et al., 2014; Christophel and Haynes, 2014; Hutzler, 2014). However, the relatively low temporal resolution of fMRI and other neuroimaging methods based on cerebral hemodynamics renders them unsuitable for decoding neural states associated with split-second decision-making. Instead, electroencephalography (EEG) or magnetoencephalography (MEG) provide methods with sufficient temporal resolution to decode neural states associated with rapid decision-making. In the present study we used MEG as our primary source of neural activity, but also conducted an fMRI study to allow for better localization of MEG activity to brain areas.

A number of studies have applied pattern classification methods to neural signals in order to decode specific cognitive states. Typically these approaches train the classifier on part (e.g., half) of the neuroimaging obtained during performance of a cognitive task and then evaluate the effectiveness of the classifier on the remaining (untrained) half of the data (Garrett et al., 2003; Wilson and Russell, 2007; Baldwin and Penaranda, 2012). This is certainly an accepted criterion for evaluating how well a particular decoding algorithm works in a particular domain of human performance. But a stricter test is necessary if such neural BCI-decoders are to be useful in a general way. The more stringent test would involve application of a trained classifier to untrained data taken from a different task in the same general cognitive domain. Such a strategy, if successful, can provide for a more generalizable test of the efficacy of neural state decoding for a given cognitive function. We used this approach in the present study by training a MEG classifier during performance of a simple flight task involving a perturbation and applying it to a more complex flight task involving similar types of perturbations during ongoing piloting. It is important to note that the ongoing piloting task uses the same control stick (controlled by articulation of the hand, wrist, and arm) as that needed to recover from the perturbation. It is therefore necessary for the BCI-decoder to be able to distinguish between brain activity responding to changes in the visual field and motor intention that are a result of piloting from brain activity responding to changes in the visual field and motor intention that arise from a perturbation (even though the BCI-decoder has not been specifically trained to do so). We additionally evaluated the generalizability of a trained model across participants' by using the weights of the model of the best participant to test performance over the trials of the complex flying task of the remaining participants.

Several MEG and EEG studies have identified neural correlates of visual and motor responses that suggest our goal of predicting motor intention to a visually presented hazard prior to actual movement is possible. Single trial response times to visual coherent motion onsets were predicted by MEG activity from 150 to 250 ms before the manual response of the observer (Amano et al., 2006). While the focus of the Amano et al. (2006) study is on the onset of visual perception, not on motor intention, it does provide a potential link between response time and the identification of the perceptual event. In a study investigating neural correlates of speeded motor responses to a visual stimulus it was found that larger low-theta complexes in EEG preceded more rapid button presses (Delorme et al., 2007). It has also been found that self-paced motor intention of reaching direction can be successfully decoded prior to movement onset (62.5 ms with 76% classification performance) using slow cortical potentials (0.1–1 Hz) recorded by EEG (Lew et al., 2014). In addition, research conducted on the detection of braking intention in simulated (Haufe et al., 2011; Kim et al., 2015) and real (Haufe et al., 2014) driving using EEG was able to make predictions about 130 ms earlier than the corresponding behavioral responses. The real-world task set out in our experiment to be able to predict motor intention to a visual hazard in the presence of complex ongoing motor control and a dynamically changing visual field goes beyond what was investigated in these previous studies. Nevertheless, we do believe that these studies taken together suggest that there may potentially be some features present in the MEG (and EEG) signal that can be decoded prior to movement onset in response to a visually presented hazard even under the robust conditions set out in our experiment.

**Figure 1:**
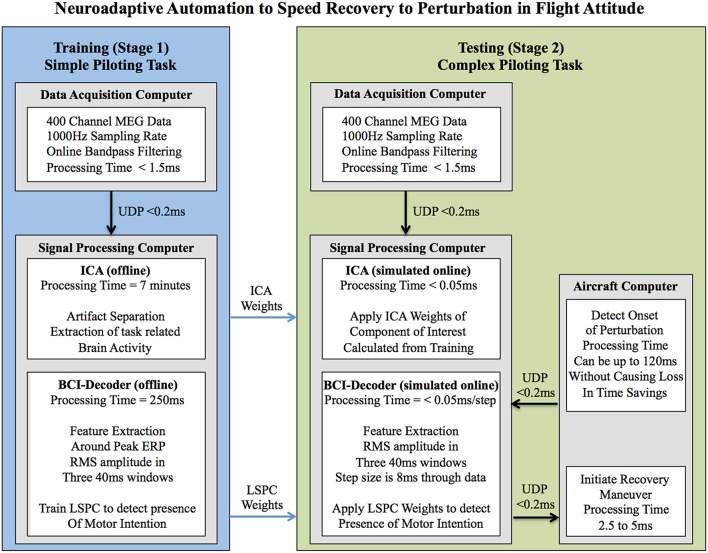
**Outline of processing procedures for the implementation of the hypothesized neuroadaptive automation to speed recovery to perturbation in flight attitude**. The goal of this system is to speed up response time for the aircraft to recover from a perturbation by decoding the motor intention of the pilot. In this way the pilot is always in control of the aircraft rather than relying on automation in which the pilot is out of the loop. It should be noted that all processing was done offline and that the online parts of the system were simulated. The processing times for each of the procedures if ran in real-time online are given. As we were carrying out an offline simulation to determine the feasibility of signal processing and the BCI-decoder performance during training and testing stages for implementation in a real-time neuroadaptive automation system the aircraft computer was not actually implemented in this study. The system is theoretically able to work in real time with only a 5–7.5 ms loss in time savings because the weights of the ICA and BCI-decoder are determined before hand and applied to the online system. The aircraft computer is a necessary part of the neuroadaptive automation system that receives commands from the BCI-decoder to implement the recovery maneuver (in this case upward elevator deflection). The aircraft computer can also send information to the BCI-Decoder that can signal the onset of potential perturbations to the airplane. This information can be used to reduce the occurrence of false-alarms made by the system (executing upward elevator deflection when there is no actual perturbation or motor intention to recover). The aircraft computer can use up to 120 ms (time of the processing window for the BCI-decoder) to determine the presence of a non-pilot initiated perturbation in attitude without causing a loss in the time savings afforded by the neuroadaptive automation system. ICA, Independent Component Anayalsis; BCI, Brain Computer Interface; LSPC, Least Squares Probabilistic Classification; UDP, Universal Datagram Protocol.

There have been previous studies (Blankertz et al., 2002; Parra et al., 2003) using online BCI to detect error-related potentials to reduce error-rate and improve overall performance. While these methods are promising they utilize data that occurs after the response is made and are thus not applicable to our objective of detecting motor intention prior to movement. It is the goal of our study to utilize an off-line BCI-decoder to evaluate the feasibility of using real-time neuroadaptive automation to enhance piloting performance by reducing response time to recover from an impending hazard (see Figure 1).

## Materials and methods

### Participants

A total of seven right–handed adults participated in this study. Five (three females and two males) were glider pilots from local university clubs. The two participants (males) that were not pilots had considerable experience with driving or flying related video games. The age of the participants ranged from 19 to 40 years with a mean of 23.9 years and SE = 2.7 years. All participants gave written informed consent for experimental procedures approved by the ATR Human Subject Review Committee in accordance with the principles expressed in the Declaration of Helsinki.

### Experimental tasks

**Figure 2:**
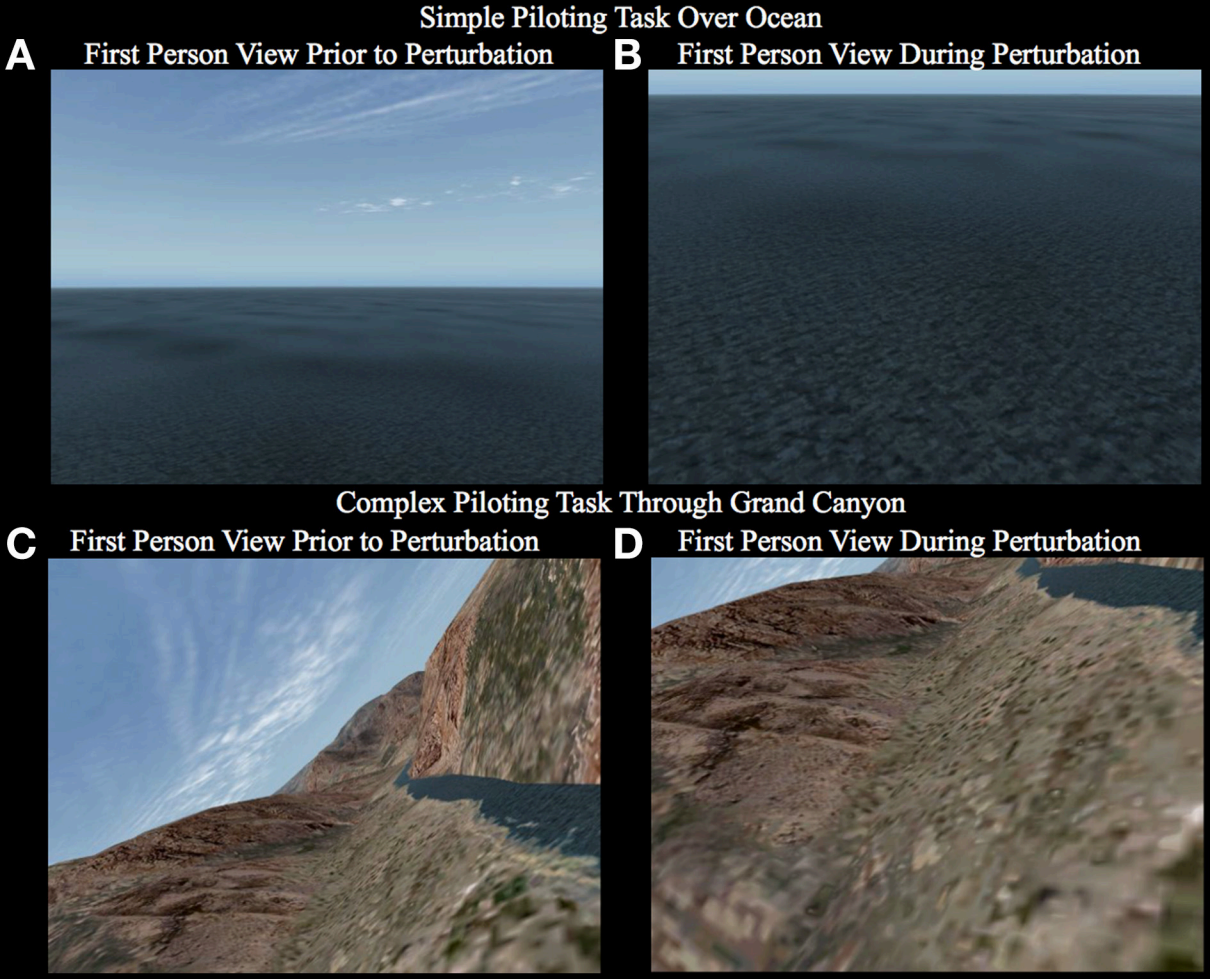
**First person view the participant observes while carrying out the simple piloting task over the ocean (A,B) and the complex piloting task (C,D)**. The first panel for each task **(A,C)** shows a representative image of what the view may appear like prior to the perturbation. The second panel for each task **(B,D)** shows a representative image of what the view may appear like during the perturbation. Notice that in the simple piloting task over the ocean **(A,B)** the bank angle is always level, whereas, in the complex piloting task the bank angle is continuously changing based on the control stick inputs to maintain the goal of tracking the river (See Supplementary Videos 1–6).

Two different tasks were used, a simple piloting task of level flight over the ocean and a more complex piloting task through the Grand Canyon. We used the first task to develop a method for decoding of neural states associated with response to a perturbation and the second task to investigate the generalizability of the method to a related but more complex situation. In both tasks the participant was given a first-person unobstructed view from the airplane (the view was as if from a camera in the front of the aircraft, see Figures 2A–D). The aircraft model simulated was an F22—Raptor using the X-plane flight simulator (Version 9.75, Laminar Research). The data for various flight parameters (elevator, aileron, rudder deflections, pitch, roll, yaw, heading, speed, dive rate, structural g-forces, latitude, longitude, altitude, etc.) and the control stick (NATA Technologies MRI and MEG compatible) deflections were collected at a mean sampling rate of 400 Hz using a UDP Matlab interface. The experimental conditions could be controlled via Matlab by using the UDP interface to give commands to the flight simulator.

#### Simple piloting task

This task had four conditions, two involving the presence or absence of a perturbation, and two in which the participant had the choice to either pilot the plane or passively watch the screen without moving the control stick [see Supplementary Videos 1–4; (1) fly_perturbation; (2) fly_noperturbation; (3) watch_perturbation; (4) watch_noperturbation; The participants viewed the 1^st^ person perspective given on the left side of the video]. The primary task required the participant to pull back on the control stick (causing an upward elevator deflection resulting in the plane to climb) as rapidly as possible in response to a perturbation in attitude (orientation of the plane with respect to the horizon) causing the plane to dive at a steep rate (see Figures 2A,B). The participant was instructed to hold the control stick but not to move it until after the perturbation occurred. The perturbation consisted of instantaneous maximum downward deflection of the elevator for 200 ms causing the plane to enter a steep dive. The trial started with the plane flying at an altitude of 107 m above sea level at a speed of 1040 kph. The perturbation occurred on 67% of the trials at a random time between 2 and 4 s (randomly determined) after the beginning of the trial. If the plane descended to 30 m above sea level the simulator was paused before the plane crashed into the ocean. At the end of each trial the simulator was paused for 1.5–2.5 s (randomly determined). The timing was the same for trials in which there was no perturbation. Before the beginning of each trial participants chose by button press whether they were going to pilot the plane or passively watch without moving the joystick. Participants were instructed to try to make about twice as many piloting trials as passive trials. The rational for having the participant select whether they were to fly or watch rather than to direct them which condition it was by instruction was to better insure that they were actually doing the task correctly. If given visual directed instructions, participants would often try to recover from the perturbation even when they were instructed to just watch. Allowing participants to choose which condition to fly or watch helped to alleviate this problem. In this study, for the simple piloting task, only the trials containing a perturbation (fly_perturbation and watch_perturbation) were used to train the BCI-decoder. Please see the section under Decoding Pilot Intention below for the rational. After the button was pressed there was a delay of 0.8–1.3 s (randomly determined) before the trial begins. The passive trials were the same as the piloting trials with the exception that the plane would pause when it reached an altitude of 30 m above the ocean, which occurred at a mean time of 1.3 s after the onset of the perturbation.

There were 90 trials per session. On average there were 40 piloting perturbation trials, 20 piloting no-perturbation trials, 20 passive viewing perturbation trials, and 10 passive viewing no-perturbation trials. The actual number of trials for each condition was dependent on the participant's choice to pilot or passively view. The percentage of perturbation trials (67%) was experimentally determined and presented randomly within each of those conditions. Each session was ~13 min. Bad trials (plane did not fly straight and level until time of perturbation) were removed from the analysis. Additionally, trials with response times slower than 700 ms from the onset of the perturbation were removed from the analysis.

#### Complex piloting task

This task involved flying through the Grand Canyon and consisted of two conditions: perturbation (67% of trials) or no perturbation (33%) [See Supplementary Videos 5, 6; (5) Grand_Canyon_perturbation; (6) Grand_Canyon_noperturbation]. Unlike the simple flying task, the participant was always required to pilot the plane. There were no passive viewing conditions. In the complex task the participant was constantly required to move the elevator and ailerons of the plane with the control stick to track the river through the Grand Canyon. The perturbation was caused by an instantaneous maximum downward deflection of the elevator for 200 ms. Depending on the attitude (particularly the angle of bank–roll) of the plane, the perturbation would cause a rapid departure from the trajectory of flight toward the ground and/or one of the cliffs (see Figures 2C,D). The plane started each trial at approximately 30 m above ground level at a speed of 1135 KPH. As in the simple task, the perturbation occurred between 2 and 4 s (randomly determined) after the beginning of the trial. There was also a pause for 1.5–2.5 s (randomly determined) at the end of each trial. Unlike the simple task, in which the participant specified by button press whether they were going to pilot the plane or passively watch, in the complex task every trial was a piloting trial. The instructions on the screen denoted that the participant could push the button when they were ready to begin the trial. After the button was pressed there was a delay of 0.8–1.3 s (randomly determined) before the trial began. Unlike the simple task, in the complex task the plane was allowed to crash into the ground or cliff. Upon a crash the system would pause the screen. There were 90 trials total in the complex piloting task. There were 60 perturbation trials and 30 no-perturbation trials. The order was randomly determined. Each session was approximately 14 min. Trials in which the plane crashed before the onset of the perturbation were removed from the analysis.

### Functional MRI

Our goal to develop a classifier of operator intention to undertake a rapid action to avoid a perturbation was to use a neuroimaging method with high temporal resolution, such as EEG or MEG. We used MEG in the current study, but in order to bolster our ability to localize MEG activity to intracortical sources, we also conducted an fMRI study of the same piloting tasks in order to establish seeds for conducting source localization analyses of MEG data. In the fMRI experiment participants underwent two sessions of the simple piloting task. Visual presentation of the flight simulation was projected by mirrors to a screen behind the head coil that could be viewed by the participant by a mirror mounted on the head coil. An fMRI compatible control stick (NATA technologies) was used by the right hand of the participant to control the elevator (back = pitch up; forward = pitch down) and aileron (roll left and right) deflections. Trigger timing of the fMRI scanning was directly read into one of the flight parameters of the flight simulator by means of a National Instruments Hi Speed USB NI USB-9162 BNC analog to digital converter.

A Siemens Verio 3T scanner was used to obtain functional T2^*^ weighted images with a gradient echo-planar imaging sequence (echo time 30 ms; repetition time 2500 ms; flip angle 80°). A total of 40 interleaved axial slices were acquired with a 4 × 4 × 4 mm voxel resolution covering the cortex and cerebellum. A single run consisted of approximately 340 scans. (The number varied depending on the randomized time and how long the participant took to make a button response to start the trial). The first three scans were discarded. Structural T2 images, later used for normalization, were also collected using the same axial slices as the functional images with a 1 × 1 × 4 mm resolution. Images were preprocessed using SPM8 (Wellcome Department of Cognitive Neurology, UCL). Echo planar images EPI were unwarped and realigned. The T2 image was co-registered to the mean EPI image. The T2 images were acquired during the same fMRI session as the EPI images with the same slice thickness. Since the head was in approximately the same position it is thought that this will facilitate coregistration. The EPI images were then spatially normalized to MNI space (3 × 3 × 3 mm voxels) using a template T2 image and the co-registered T2 image as the source. Normalization was done using the T2 image rather than EPI because we believe it gives better results due to better spatial resolution. The images were smoothed using an 8 × 8 × 8 mm FWHM Gaussian kernel. Regional brain activity was assessed using a general linear model employing an event-related analysis in which the onset times were convolved with a hemodynamic response function. High pass filtering (cutoff period 128 s) was carried out to reduce the effects of extraneous variables (scanner drift, low frequency noise, etc.). Auto-regression was used to correct for serial correlations. The six movement parameters were used as regressors of non-interest in the analysis to account for biases in head movement correlations present during the experimental conditions. Anatomical T1 weighted images were acquired with a 1 × 1 × 1 voxel resolution for use in constructing source models for localizing brain activity recorded by MEG.

### MEG

In the MEG experiment participants underwent three sessions of the simple piloting task and one session of the complex piloting task. The first two sessions of the simple piloting task were used for training the decoding algorithm. The third session of the simple piloting task was used to evaluate the effectiveness of the trained algorithm in decoding neural states when participants perform the same task. As discussed previously, however, an effective classifier should be able to decode not only neural states on the same task that it has been trained on, but on more complex versions of the task that the classifier has not been trained on—that is, whether the classifier can achieve transfer. Accordingly, we also assessed the effectiveness of the classifier in decoding neural activity preceding detection and response to a perturbation in the complex piloting task. Visual presentation of the flight simulation was projected to a mirror to a screen above the participant's head. An fMRI compatible control stick (NATA technologies) was used by the right hand of the participant to control the elevator (back = pitch up; forward = pitch down) and aileron (roll left and right) deflections. Trigger timing for the start of each trial and the start of the perturbation was registered by a photodiode placed on the screen. A small white square was constantly presented on the lower center part of the screen (out of the view of the participant) at the start of each trial and at the onset of the perturbation the small square turned black for 20 ms. The light intensity change was detected by the photo diode and written directly to one of the extra channels on the MEG.

**Figure 3:**
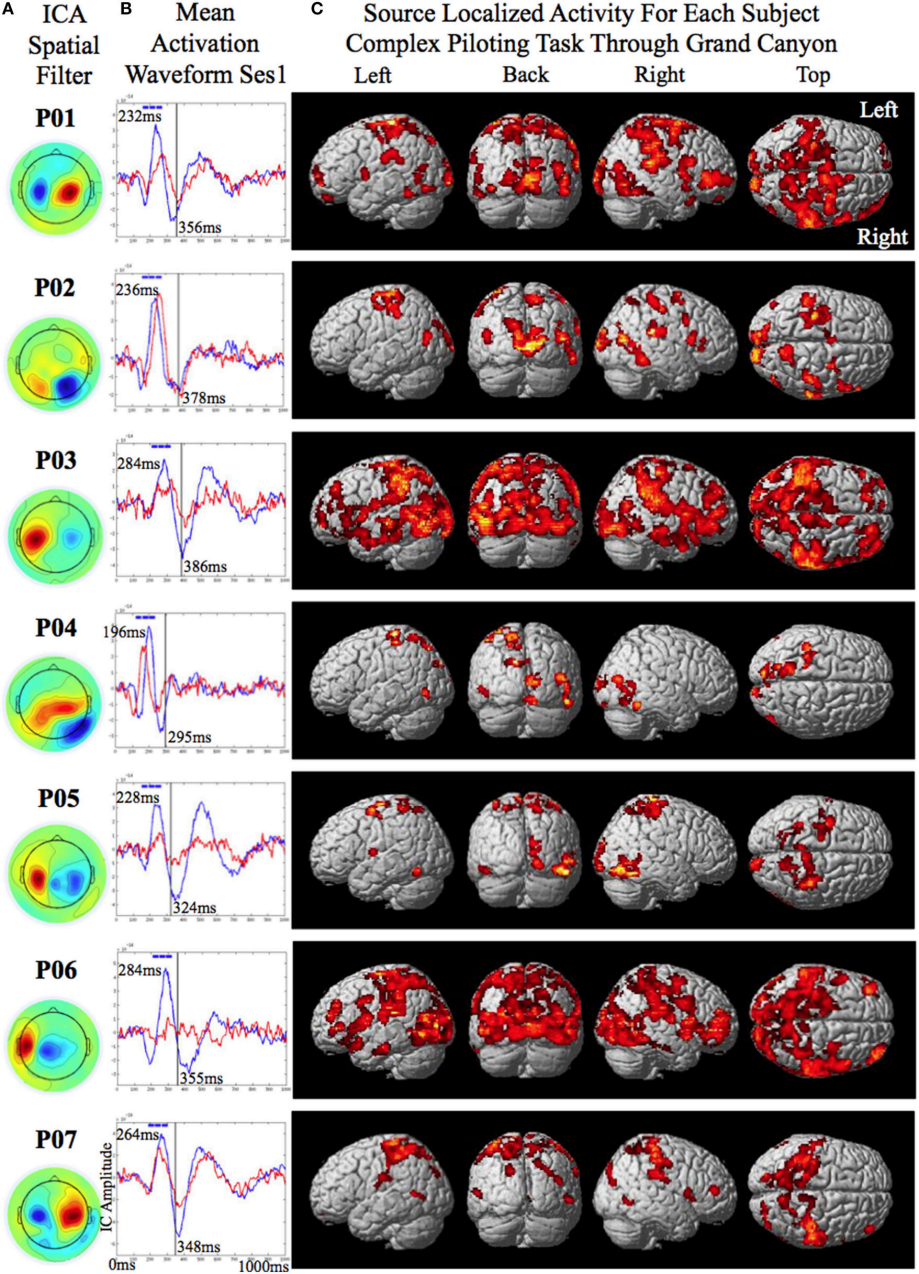
**Source localized activity for each participant (P01–P07 denotes participant identification number). (A)** On the left the independent component analysis spatial filters for the MEG channels are shown for each participant. **(B)** The mean activation waveform for session one with the peak latency given in the upper corner for each participant. The blue boxes over the peak denote the three 40 ms windows the decoder was trained on. The mean response time is denoted by the gray line in the plot. The corresponding value is shown to the bottom right of this line for each participant. **(C)** The estimated current using variational Baysian multimodal encephalography VBMEG is shown rendered on the surface of the brain for each participant.

The data was recorded using a Yokogawa 400 channel MEG supine position system. Head movement was restrained by using a strap across the forehead. A sampling rate of 1000 Hz was used with input gain of × 5 and an output gain of × 100. The trials were segmented 1000 ms before and after the onset of the perturbation. For trials with no perturbation the timing of the virtual perturbation was given by the photodiode and used as the onset point for segmentation. The data were down sampled from 1000 to 250 Hz and filtered using a causal Butterworth online bandpass filter from 2 to 100 Hz. Only bad trials in which there was a machine failure in the flight simulator causing the plane to verge from a straight and level course (for the simple piloting task) or bad trials in which the plane crashed before the onset of the perturbation (for the complex piloting task) were removed from the data. Besides bandpass filtering there was no manual or automated artifact cleaning of the data prior to independent component analysis. Infomax independent component analysis (EEGLAB, Delorme and Makeig, 2004) with principal component analysis PCA reduction to 64 components was conducted on the first two sessions of the simple piloting task (processing time was approximately 7 min). ICA has been shown to be well suited for separation of artifact and task related components (Delorme et al., 2007). The weights derived from the ICA were used to calculate component activation waveforms for the trials in sessions one and two. They were also used to calculate component activation waveforms for the trials in sessions three of the simple piloting task and the session of the complex piloting task. There were two reasons that the weights from the first two sessions were used to calculate the activation waveforms of the later sessions: first, we did not want to bias the classification results of the BCI-decoder used for training by including the test data of the later sessions; and second, we wanted to simulate conditions required to run the BCI-decoder as if we were running it online in real-time. The independent components showing evoked responses to the averaged perturbation piloting trials were considered for training of the BCI-decoders. Each participant had one evoked potential related component with an ICA spatial filter showing a prominent sinc and source (See Figures 3A,B). All preprocessing steps described above were automated except for the selection of the independent components showing evoked responses, which was done by visual inspection of the averaged activation waveform and the ICA spatial filter. This step can also be automated if desired.

### MEG source localization analysis

Source localization analysis involves the following steps: (1) Determining the position of the head (brain) within the MEG device, (2) Segmentation of the cortex of the brain, (3) Estimation of the leadfield model on the vertex points of the segmented cortex, and (4) Current source estimation on the cortex.

1. Five coils attached to the participant's head (one behind each ear, and three across the forehead) were used to determine the position of the head within the MEG. The positions of the five coils on the participant's head were measured by the Polhemus FastSCAN Cobra system. This system allows for a 3D laser scan of the face as well as the coordinate location for the five markers to be obtained. Matlab software (part of the VBMEG toolbox) was used to register the coordinate space of the 3D face image to the participant's anatomical T1 MRI structural image. Once the position of the five coils in reference to the MEG sensors are known the position of these sensors can be registered in the coordinate space of the participant's T1 MRI structural image.
2. The cortex was segmented from the brain using FreeSurfer software (Fischl et al., 1999). This software was also used to make an inflated model of the cortex used for display.
3. The segmented vertex points of the cortex were used to estimate the leadfield matrix using the Sarvas equation (Sarvas, 1987).
4. Current source on the surface of the cortex was estimated using variational Bayesian multimodal encephalography (VBMEG) that uses fMRI information as a prior constraint in the analysis. See the articles by Sato et al. (2004) and Yoshioka et al. (2008) for a detailed description as well as the tested accuracy of source localization for VBMEG.

The VBMEG analysis used the fMRI *t*-values of the contrast of the perturbation piloting condition vs. the no perturbation piloting condition on the simple piloting task. The results of the SPM analysis for the contrast for each participant (using a threshold of *p* < 0.05 uncorrected, with a spatial extent of 50 voxels, and masking out activity in the cerebellum and subcortical areas) were projected onto the brain of their own T1 image using their individual specific normalization parameters. For one of the participants for which fMRI data was not collected the random effects analysis across all of the participants for the same contrast (using a threshold of *p* < 0.05 uncorrected, with a spatial extent of 50 voxels, and masking out activity in the cerebellum and subcortical areas) was used as a prior and projected back to the individuals T1 image using their specific normalization parameters. The fMRI *T*-values were then mapped to the vertex points of the segmented brain serving as prior information for the VBMEG analysis. A lenient uncorrected threshold of *p* < 0.05 was used to ensure that sufficient vertex points of the brain were given prior information for the VBMEG analysis. Using a conservative threshold corrected for multiple comparisons for the fMRI analyses may considerably restrict the extent of prior information for the VBMEG analysis.

The activation waveforms of the trials from all conditions and sessions for both the simple and complex tasks were projected to the MEG sensor space (400 channel) using the weights of the independent components as determined from the ICA on the trials from the first two sessions of the simple piloting task. The mean activity of the trials for each condition was used in the VBMEG analysis. The noise model, serving as a baseline, was calculated using the activity from the no perturbation passive viewing condition. The VBMEG analysis estimated current activity over the entire cortex using a variance magnification factor = 500 and a confidence parameter = 500 [these parameters are such that they give somewhat less weight to the fMRI prior activity in determining the location of the source activity Sato et al. (2004) and Yoshioka et al. (2008)]. The time period for current estimation was 250 samples and the time step for the next period was 100 samples. The output of the analysis was the mean estimated current across trials for each cortical vertex point for each condition.

To determine the location of current on the brain thought to underlie the response to the perturbation and to be able to compare the results across participants the following procedure was used for each participant using data from the complex piloting task: For each of the vertex points (there were ~6000 for each participant), the root-mean-squared RMS current was determined for perturbation recovery and for a baseline period prior to perturbation: The RMS current for perturbation recovery was calculated from 12 ms before and 8 ms after the new mean response time (utilizing performance of the adaptive automation BCI-decoder—see Results section). The RMS current for the baseline period was calculated from 400 ms before onset of the perturbation to just before the onset of the perturbation. The current for each vertex point was normalized by subtracting 20 times the mean RMS current of the baseline period (across all vertex points) from the RMS current of perturbation recovery for each vertex point and then dividing by the maximum RMS current across all vertex points. The normalized current of the vertex points that were greater than zero were projected to the standard template brain (2 × 2 × 2 mm) (given in SPM8) using the MNI coordinates determined during segmentation by Free Surfer. The resulting images were smoothed using a FWHM 8 × 8 × 8 mm Gaussian kernel. Because smoothing may cause activity to be spread to regions that were not originally active a threshold was used such that only voxels showing mean RMS values greater than the lowest value of the original smoothed voxels (corresponding to the original projected vertex points) were considered to be significant (using a spatial extent threshold of 100 voxels). The intersection of active voxels across all seven participants was used to define common activity. The SPM Anatomy Toolbox v1.8 (Eickoff et al., 2005) was used to determine the labels of active brain regions.

### Decoding pilot intention

We developed a method to decode the participant's intention to perform an action in response to a perturbation by training a classifier on neural data taken from the first two sessions of the simple piloting task. The classifier was then evaluated by testing its ability to decode participant intention on the third session of the same task. As a more stringent test of classifier performance—an examination of its transfer generalizability—we then examined its ability to decode intention in the complex piloting task. It should be noted that this classifier represents an open loop simulation of a BCI in order to test the feasibility of such a method for real-time implementation of neuroadaptive automation using a closed loop BCI-decoder. See Figure 1 for a depiction of the hypothesized neuroadaptive automation system implemented in this study.

The training of the classifiers was conducted using trials from the first two sessions of the simple piloting task. The two classes to be decoded were presence of perturbation while piloting the plane vs. presence of perturbation while passively viewing. The reason for selecting these contrasts to train on was because we wanted to ensure that the BCI-decoder was not just picking up the visual evoked response induced by the perturbation but was extracting activity related to the attentional components of the response to the perturbation in relation to the intention to recover from the change in attitude. Rather simple features were used for decoding in the hope that they would generalize across sessions, tasks, and participants. The first step in calculating the features used for decoding was to determine the time point of the absolute peak in the mean evoked response (that was less than 300 ms) to the onset of the perturbation in the selected independent component of the perturbation piloting trials of the first session of the simple task used for training (the peak time for the participants was as follows: S1 = 232 ms; S2 = 236 ms; S3 = 284 ms; S4 = 196 ms; S5 = 228 ms; S6 = 284 ms; S7 = 264 ms; mean = 246 ms) (See Figure 3B for the mean activation waveforms for the Fly and Watch conditions for each participant from session 1). For each trial RMS amplitude was calculated within two consecutive 40 ms windows prior to the time of the peak of the averaged evoked potential and one 40 ms window after (These windows are depicted as blue bars at the top of the mean activation waveforms in Figure 3B for each participant). The RMS amplitude values in these three windows served as the features for decoding for the perturbation piloting trials. To help in generalization and to bias the classifier to make fewer false alarms the perturbation passive trials used three separate time points to extract the features (120, 60, and 0 ms before the onset of the perturbation). This had the effect of increasing the number of training trials for the perturbation passive condition by three. Since there were originally half as many perturbation passive trials than perturbation piloting trials this increased the training ratio to be about 1.5 times as many trials for the perturbation passive condition to that of the perturbation piloting condition. The greater number of training trials and the greater variability for the perturbation passive condition is used to increase the ability to reject trials that are not from the perturbation piloting condition (reduce false-alarms) and increase the noise variability with regards to timing such that the classifier may more readily generalize to the complex task in which the attitude of the plane (and thus the visual image on the screen) is constantly changing as a result of the continuous piloting task of tracking the river in the Grand Canyon. The reason we did not use the no perturbation piloting task as one of the conditions to train the BCI-decoder on is that it would likely just extract the visual evoked response to the perturbation piloting task and not extract the attention related component of the motor intention for attitude recovery that we are interested in determining. Given the continuous changes in attitude of the plane while maneuvering on the complex task a BCI-decoder that is based on visual evoked perturbations from the simple task may result in a large number of false alarms.

The BCI-decoder was trained on approximately 80 trials of the perturbation piloting task and 120 trials of the perturbation passive task using the Matlab Least Squares Probabilistic Classification (LSPC) toolbox (Sugiyama et al., 2010). LSPC uses a linear combination of kernel function to model the class-posterior probability. Regularized least-squares fitting of the true class-posterior probability is used to learn its parameters (Sugiyama et al., 2010). The use of least-squares fitting to determine a linear model allows for a global solution to be made analytically providing a considerable speedup in computational time. The default parameters were used in training of the LSPC models (see Matlab code: Sugiyama et al., 2010). The time required to train the classifier is approximately 0.25 s. Prior to training the features for the trials were normalized by subtracting the mean and dividing by the standard deviation. The mean and standard deviation from the training trials were used to normalize the trials used for testing. The first test set consisted of trials from the session of the simple piloting task that was not used during training. There were approximately 40 perturbation piloting trials and 20 perturbation passive trials to be classified using the train LSPC model. Balanced accuracies (Brodersen et al., 2010) are reported to account for biases in unequal number of trials in the two conditions to be classified. The test data consisted of features computed at the time point specified by the peak of the evoked response determined from the training data. No information about the distribution of the test data was used. The BCI-decoder treats each test trial as independent. One hundred BCI-decoders were trained and then tested using trials from the simple piloting task. The primary parameter that is random for training of the model for each BCI-decoder is the order of the trials in the training cross validation steps. The BCI-decoder with the best performance as determined by balanced accuracy was used to test the trials from the complex piloting task.

The goal for the BCI-decoder in the complex piloting task was to be able to detect the intention to recover from a perturbation in attitude faster than by movement of the control stick by the hand. The selected LSPC model trained on the simple piloting task was used for testing of the complex piloting task. Additionally the same parameters (mean and standard deviation) used during training were also used on the test set for normalization of the features. For the perturbation piloting trials and the no perturbation piloting trials the LSPC model began testing at time point zero when the perturbation started. The window for the BCI-decoder was 120 ms encompassing the three 40 ms time windows in which the RMS amplitude was calculated. Therefore, the earliest time the perturbation could be detected was at 120 ms. The 120 ms time window tested by the BCI-decoder was incremented in 8 ms steps through 1000 ms of the data for each trial. The earliest point at which the BCI-decoder detected the presence of a perturbation piloting trial was the point at which the adaptive automation would be implemented to recover attitude. The time between detection by the BCI-decoder and the onset of the control stick by the hand to recover from the perturbation in attitude was used to evaluate the time benefit (time savings) of the implementation of the adaptive automation. The trial was only considered a hit if the BCI-decoder predicted time was faster than the actual movement time of the control stick. To determine the statistical significance of the BCI time savings, BCI-decoders were trained using 1000 random permutations of the labels and each was tested on the complex piloting task. All 1000 permuted models used for evaluation had less than 25% false positives. This criterion was used in order to be comparable to the false positive performance of that of the BCI models trained with correct labels. The perturbation time benefit were calculated for each of the 1000 permuted models and used as a distribution to compare against the model trained with the actual labels.

In order to evaluate the generalizability of a single model across participants the weights of the model of the participant with the best performance were used to test the trials of the complex flying task of the remaining six participants. Performance measures including BCI time savings were determined using the same method as applied when using each participants corresponding model to test the trials of the complex piloting task (see above).

### Procedure

Participants underwent the fMRI and MEG sessions on separate days. The fMRI experiment was used to calculate a prior for the MEG source localization analysis using Variational Bayes Multimodel Encephalography (VBMEG). Six of the seven participants participated in the fMRI experiment. One participant only did the MEG experiment. All of the participants that participated in the fMRI experiment did it prior to the MEG experiment. MRI anatomical T1 scans were acquired for all seven participants and used to make models for source localization analysis using VBMEG. All analyses were conducted using Matlab software unless otherwise stated.

## Results

### Behavioral performance

The response times (RTs) for each of the participants to initiate pull back on the control stick in reaction to a perturbation causing the plane to enter a steep dive for both the simple piloting task and the complex piloting task are presented in Table 1. The RT in the complex task (median = 436.5) was not found to be significantly higher than in the simple task (median = 368.5), *p* = 0.0781 (df = 6). However, there is a tendency in this direction. The number of trials the plane crashed into the ground/cliff, as well as the number of bad trials (resulting from machine failures and/or movement before the onset of the perturbation for the simple task and crashes before the onset of the perturbation for the complex task) are also presented in Table 1. As Table 1 indicates, these numbers were relatively small, but were greater in the complex task. It should be noted that bad trials were excluded from analysis and not used for calculation of response times or training/testing of the BCI-decoders. In some cases on the complex task there were crashes after the onset of the perturbation. These trials were not excluded from analysis.

**Table 1:** **Response time to pull back on control stick after start of perturbation for the two training sessions and two test sessions**.

**ID**	**RT train Ses1 (ms)**	**BT train Ses1**	**RT train Ses2 (ms)**	**BT train Ses2**	**RT test simple (ms)**	**BT test simple**	**RT test complex (ms)**	**BT test complex**	**CT test complex**
1	356.4	1	387.7	2	370.4	1	357.9	0	0
2	377.7	0	427.0	2	452.6	3	436.5	8	9
3	386.5	2	371.4	0	384.4	0	454	2	6
4	295.1	0	303.7	0	305.5	0	359.8	2	6
5	323.9	0	336.9	0	342.3	0	462.2	1	1
6	355.0	0	386.3	9	368.5	3	480.4	5	0
7	348.4	0	347.1	0	337.8	0	424.1	4	9
Group mean	349.0	0.43	365.7	1.86	365.9	1.0	425.0	3.14	10.33

*ID, Participant identification number; RT, Response Time; Ses, Session; BT, Bad Trial; CT, Crash Trial*.

### Source localization

**Figure 4:**
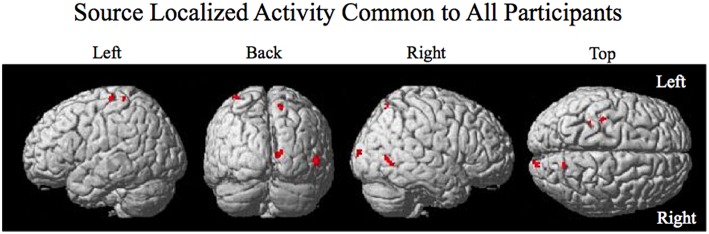
**Source localized activity common to all participants**. Activity is present in the left pre- central gyrus, the left post central gyrus, the right superior parietal lobule, the right primary visual cortex V1, and the right visual motion processing area V5.

The smoothed RMS current centered around the time of the perturbation on the complex piloting task of the activation waveform of the projected task related independent component rendered on the surface of the brain (See Methods for details of source localization analysis) is displayed for each participant along with the corresponding independent component spatial map in Figure 3C. There was some degree of variability in the extent to which different brain regions were active across participants (Figure 3C, Table 2). As can be seen in Figure 4 and Table 3 brain regions that were commonly active for all participants include the pre-central gyrus (including premotor cortex), post- central gyrus, the superior parietal lobule, the primary visual cortex, and human occipital cortex visual motion processing area V5 (hOC5). It should be noted that while source localization is interesting in determining the brain regions associated with the independent component upon which decoding is made it is not a necessary step in implementation of the proposed neuroadaptive automation brain-machine interface.

**Table 2:** **MNI coordinates of clusters of brain activity for each participant**.

**Brain region**	**P1 MNI**	**P2 MNI**	**P3 MNI**	**P4 MNI**	**P5 MNI**	**P6 MNI**	**P7 MNI**
Orbital Gyrus	−22,58, −4		−34,56, −6			26,45, −13	−6,56, −14
	−12,54, −16		48,36, −10				22,46, −12
SFG, MFG			−14,44,32			−38,40,30	
			−32,40,36			42,51,7	
IFG BA44	−52,4,10	54,14,10	−49,5,6			−50,10,6	54,18, −2
	56,16,22	56,8,34				58, −1,7	
IFG BA45	45,44,2	58,30,0	−50,25, −1			−48,36,4	48,44,12
						56,26,2	
SFG SMA BA6	1, −16,66	−4,0,58	−4, −3,68				
			11, −13,67				
PMC BA6	−35, −25,68	−34, −23,68	−30, −27,68	−36, −28,68	−38, −8,64	−26, −21,70	−33, −25,67
	46, −8,48	28, −8,64			22, −31,76	58,4,31	
Pre−CG BA4	−48, −16,56	−35, −25,58	−35, −25,54	−32, −28,69	−42, −18,54	−9, −42,75	−30, −38,70
	42, −10,46		12, −27,74		8, −34,76	−28, −27,59	
					38, −30,61	9, −38,75	
Post−CG BA1,2,3	−18, −36,76	−24, −36,72	−40, −41,61	−30, −34,70	−30, −44,64	−35, −35,67	−40, −44,58
	−48, −30,58	−52, −32,54	44, −22,60	−44, −34,58	−62, −8,10	−47, −26,55	56, −20,46
		46, −32,60			38, −32,60	53, −24,55	44, −30,60
						62, −24,26	
SPL	−10, −76,52	10, −70,56	−38, −48,57	−14, −54,66	−24, −60,62	−16, −74,48	−16, −70,62
	−22, −82,48	24, −56,56	−8, −65,58	−10, −90,34	20, −66,60	18, −66,48	−16, −86,38
	12, −56,68		9, −64,61	16, −68,56			13, −66,62
	26, −50,66		41, −45,58				
IPC	−54, −70,16	−46, −74,18	−52, −44,38			−58, −39,35	−58, −18,28
	−56, −24,30	46, −70,14	48, −43,35			57, −34,40	60, −30,30
	60, −22,34	68, −30,20	58, −30,40				46, −82,20
hOC5 (V5) MT, IOG	52, −67, −1	−44, −74,18	−40, −70, −2	−52, −72, −2	52, −62, −16	−44, −72,5	−52, −70,0
		56, −62,4	54, −67,13	50, −70,0		53, −69,1	56, −64, −2
hOC4	−38, −72, −12		32, −71, −1		46, −80, −15	−32, −77,0	
						40, −72, −12	
Cuneus Calcarine Gyrus BA17,18	−12, −104,4	−4, −72,2	−8, −68,4	−2, −80, −2	16, −94,16	−10, −98,2	−4, −72,0
	−24, −102, −6	−10, −94,26	−8, −94, −8	8, −82,10	20, −98, −10	12, −90,0	10, −70,0
	20, −98,22	−10, −100,8	18, −92, −14	12, −102,4		6, −86,16	
	14, −98,0	10, −70,2					
	16, −100, −14	16, −96,20					
MTG	−56, −64, −2		−48, −24, −10		55, −66, −2		
			−56, −59,8				
			60, −26, −16				
			54, −63, −2				
ITG	−54, −50, −18	60, −48, −12	−46, −60, −10	54, −54, −20	50, −56, −16	−44, −63,0	
	48, −50, −12		−52, −32, −20		−44, −62, −16	−56, −20, −24	
			−58, −24, −20			60, −46,0	
Temporal Pole			−50,6, −15			−48,12, −20	
						58, −2, −6	

*P, participant identification number; IFG, Inferior Frontal Gyrus; SFG, Superior Frontal Gyrus; SMA, Supplementary Motor Area; PMC, Premotor Cortex; Pre-CG, Pre Central Gyrus; Post-CG, Post Central Gyrus; SPL, Superior Parietal Lobule; IPC, Inferior Parietal Cortex; hOC5 (V5), Human Occipital Cortex Visual motion processing area V5; MT, Middle Temporal Cortex overlaps area hOC5; IOG, Inferior occipital gyrus; MTG, Middle Temporal Gyrus; ITG, Inferior temporal gyrus. MNI coordinates of Clusters of root-mean-squared RMS current 12 ms before and after the mean time in which the decoder detected motor intention to the presence of a perturbation. The threshold of significant RMS current activity at a specific vertex point was set at 20x the mean baseline RMS current from −400 to 0 ms across all vertex points. A spatial extent threshold of 100 voxels was used on the smoothed projection into MNI space*.

**Table 3:** **MNI coordinates of clusters of brain activity common across all participants**.

**Brain region**	**All seven participants MNI**
Pre-CG, PMC BA4,6	−36,−26,68
Post-CG BA1,2,3	−30,−38,68
SPL 7P 7A Precuneus	12 −68,60
Cuneus (V1) BA17	16,−96,10
hOC5(V5)	52,−62,0

*Pre-CG, Pre Central Gyrus; Post-CG, Post Central Gyrus; SPL, Superior Parietal Lobule; hOC5 (V5), Human Occipital Cortex Visual motion processing area V5*.

*MNI coordinates of Clusters of root-mean-squared RMS current 12 ms before and after the mean time in which the decoder detected motor intention to the presence of a perturbation that are common across all seven participants. The threshold of significant RMS current activity at a specific vertex point was set at 20x the mean baseline RMS current from −400 to 0 ms across all vertex points*.

### BCI-decoder performance

The results of the performance of the BCI-decoder are presented in Tables 4–**9**. The performance of the best (as determined by the highest balanced accuracy score) out of 100 BCI-decoders tested on the novel sessions of the simple piloting task is presented in Table 4 for each participant. The average over all 100 BCI-decoders is given in the table for comparison. The BCI-decoder for six of the seven participants showed significant differences (*p* < 0.05) in being able to distinguish between perturbation piloting trials and perturbation passive viewing trials. The mean balanced accuracy performance was approximately 70%. Certainly the selection of the best BCI-decoder out of 100 trained biases these results, however, it was our goal to find the model that may best extract attentional information related to the intention of recovering from the perturbation in attitude. In this respect we feel justified in selecting the best model trained and tested on the simple piloting task to use for testing in an unbiased manner on the complex piloting task. Although training the BCI-decoder to distinguish between the perturbation piloting and no perturbation piloting trials on the simple piloting task may have provided better performance when testing on the novel session from the same task it is likely that the model would have learned the response to the visual aspects of the perturbation rather than the neural activity related to the intention to recover attitude.

**Table 4:** **Novel test session classification performance: simple piloting task over ocean: detect perturbation piloting vs. perturbation passively watch**.

**ID**	**bacc_mean**	**bacc_ppi1**	**bacc_ppi2**	**bacc_p**	**tp**	**fn**	**fp**	**tn**	**hr**	**far**	**d′**	**a′**
1	61.5 (58.4)	48.6	73.5	0.039	21	16	6	13	0.57	0.32	0.65	0.70
2	58 (51.2)	45.9	69.3	0.093	15	21	5	16	0.42	0.24	0.50	0.67
3	73.1 (72.4)	61.1	83.0	0.023	26	15	2	14	0.63	0.13	1.49	0.85
4	73.8 (67.1)	62.2	83.7	0.009	27	13	3	16	0.68	0.16	1.46	0.85
5	76.3 (75.0)	67.3	84.4	0.0002	23	17	0	19	0.58	0.05	1.86	0.89
6	81.1 (78.8)	70.9	89.4	0.0001	27	10	1	18	0.73	0.05	2.23	0.91
7	64.8 (62.6)	52.9	75.6	0.009	21	19	4	16	0.53	0.20	0.90	0.76
Group mean	69.8 (66.5)	58.4	79.8	0.016	23	16	3	16	0.59	0.16	1.30	0.80

*ID, Participant identification number; bacc_mean, Balanced accuracy mean in percent; bacc_ppi, Posterior probability intervals; bacc_p, p value; TP, true positive (hit); FN, false negative (miss); FP, false positive (false alarm); TN, true negative (correct rejection); HR, hit rate; FAR, false alarm rate; d′, d prime; a′, a prime*.

*In the case when the FAR = 0 calculation of FAR is made by adding 1 to the original FP and TN values*.

*The performance scores are for the best out of 100 BCI-decoders trained on the first two sessions and tested on the novel simple piloting task session. The average balanced accuracy for all 100 BCI-decoders is given in parentheses for comparison*.

*The bacc_p value for the group mean is the Wilcoxon signed rank test over the bacc_mean values for the 7 subjects that the values are greater than 50*.

As discussed above the model with the highest balanced accuracy on the test session of the simple piloting task was used to test the session of the complex piloting task. The goal was to simulate the use of a brain computer interface in real time that would initiate the use of adaptive automation to initiate recovery from a perturbation in attitude faster than could be done by moving the control stick by the hand. In accomplishing this goal the BCI-decoder was used on a 120 ms window starting at the time of the perturbation and stepping through the data in 8 ms steps. The BCI-decoder was also tested on trials in which there was no perturbation within the same time region in which the perturbation may have occurred. This point was determined randomly during the experiment and triggered on the MEG trace using a photodiode (see Methods). Bad trials were eliminated from the analysis (see Table 1). The first instance of the classification by the BCI-decoder as a perturbation piloting trial is the time point at which the adaptive automation is initiated. Only trials in which the BCI-decoder is faster than the movement of the controls stick are counted as hits (true positives). The results of the classification performance for the complex piloting task are presented in Table 5. Because unequal number of trials existed for perturbation piloting and no perturbation piloting trials balanced accuracies were used (Brodersen et al., 2010). All seven participants showed significant classification performance above chance even with the additional criteria that the detection of a perturbation piloting trial had to be *before* movement of the control stick. In these cases where there was classification of a perturbation trial after control stick movement, the trial was counted as a miss (false negative). The ratio of correct rejections (true negatives) to false alarms (false positives) was greater than the ratio of hits (true positives) to misses (false negatives). The mean balanced accuracy across participants was 73%. Table 6 shows the performance on the complex piloting task of the six subjects tested using the weights from the model of the best participant. The results indicate that the balanced accuracies of all six participants showed significant classification performance above chance (Table 6). While there were significant differences in hit rate and false alarm rate between the generalized and own model tests there were no significant differences, using the Wilcoxon signed rank test, in the primary performance measures including balanced accuracy, d′, and a′ (a′ a prime or area under the curve was calculated by the method given in Macmillan and Creelman (1991) (See Table 3B).

**Table 5:** **Novel test session classification performance: complex piloting task through Grand Canyon: detect perturbation piloting vs. no perturbation piloting**.

**ID**	**bacc_mean**	**bacc_ppi1**	**bacc_ppi2**	**bacc_p**	**tp**	**fn**	**fp**	**tn**	**hr**	**far**	**d′**	**a′**
1	85.6	78.3	91.5	0.0001	47	13	1	29	0.78	0.03	2.62	0.93
2	66.6	58.1	74.6	0.02	22	30	2	28	0.42	0.07	1.31	0.81
3	74.7	65.5	82.7	0.0001	38	20	4	26	0.66	0.13	1.51	0.85
4	76.7	67.1	85.1	0.0001	46	12	7	23	0.79	0.23	1.55	0.86
5	66.1	56.3	75.1	0.001	32	27	6	24	0.54	0.20	0.95	0.76
6	79.4	70.1	87.4	0.0001	45	10	6	24	0.82	0.20	1.75	0.88
7	63.7	54.5	72.2	0.003	24	32	4	26	0.43	0.13	0.93	0.76
Group mean	73.3	64.3	81.2	0.016	36	21	4	26	0.63	0.14	1.52	0.84

*ID, Participant identification number; bacc_mean, Balanced accuracy mean in percent; bacc_ppi, Posterior probability intervals; bacc_p, p value; TP, true positive (hit); FN, false negative (miss); FP, false positive (false alarm); TN, true negative (correct rejection); HR, hit rate; FAR, false alarm rate; d′, d prime; a′, a prime*.

*The bacc_p value for the group mean is the Wilcoxon signed rank test over the bacc_mean values for the seven participants that the values are greater than 50*.

**Table 6:** **Generalization of performance using best subjects weights: novel test session classification performance: complex piloting task through Grand Canyon: detect perturbation piloting vs. no perturbation piloting**.

**ID**	**bacc_mean**	**bacc_ppi1**	**bacc_ppi2**	**bacc_p**	**tp**	**fn**	**fp**	**tn**	**hr**	**far**	**d′**	**a′**
1	–	–	–	–	–	–	–	–	–	–	–	–
2	57.7	51.9	63.8	0.007	9	43	0	30	0.17	0.03	0.91	0.79
3	73.8	64.9	81.6	0.0001	35	23	3	27	0.60	0.10	1.54	0.85
4	68.6	60.3	76.3	0.0001	27	31	2	28	0.47	0.07	1.41	0.82
5	63.4	54.8	71.4	0.002	23	36	3	27	0.39	0.01	1.00	0.77
6	80.8	71.9	88.3	0.0001	43	12	4	26	0.78	0.13	1.89	0.89
7	64.3	56.0	72.0	0.0007	21	35	2	28	0.38	0.07	1.18	0.79
Group mean	68.1 (71.2)	60.0 (61.9)	75.6 (79.5)	0.032 (0.032)	26^*^ (35)	30^*^ (21)	2^*^ (5)	28^*^ (25)	0.47^*^ (0.61)	0.07^*^ (0.16)	1.32 (1.33)	0.82 (0.82)

*ID, Participant identification number; bacc_mean, Balanced accuracy mean in percent; bacc_ppi, Posterior probability intervals; bacc_p, p value; TP, true positive (hit); FN, false negative (miss); FP, false positive (false alarm); TN, true negative (correct rejection); HR, hit rate; FAR, false alarm rate; d′, d prime; a′, a prime*.

*The bacc_p value for the group mean is the Wilcoxon signed rank test over the bacc_mean values for the six subjects that the values are greater than 50*.

*The number in parentheses are the group mean values of the original decoder excluding sub01*.

* *Denotes p < 0.05 on paired Wilcoxon signed rank test for the comparison between the original decoder and the one trained with sub01 model over the six participants*.

**Figure 5:**
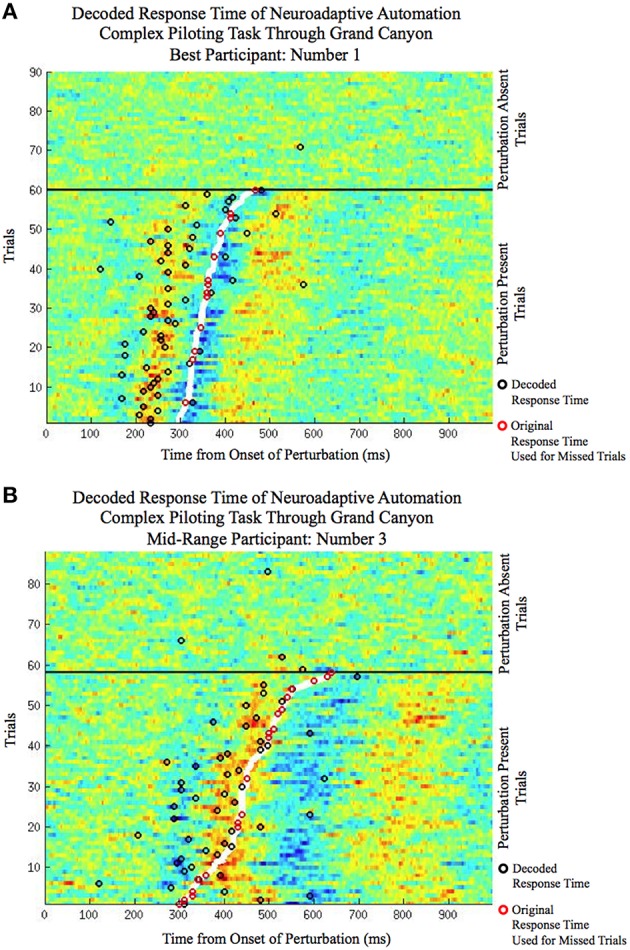
**The decoded response time (circles) plotted on the single trial activation waveforms (ranging from: red: large positive amplitude to blue: large negative amplitude) of the selected independent component of the simulated neuroadaptive automation on the complex piloting task for (A) the best participant (P01) and (B) the middle range participant in terms of classification performance (P03)**. Both perturbation absent trials (top of each plot) and perturbation present trials (bottom of each plot) are shown. The perturbation present trials are arranged in order of fastest manual response time (bottom) to the slowest (top). The manual response times are denoted by the thick white line for the perturbation present trials. The decoded response time, by the simulated neuroadaptive automation (BCI classifier), of each trial is denoted by a black circle. For perturbation present trials the black circles denote hits when their time is faster than the manual response time (white line). For perturbation absent trials the black circles denote false alarms. A red circle is shown over the original response time in the case when the simulated neuroadaptive automation failed to classify the trial as a hit (misses) or in which it was slower than the original response time (slow responses).

The improvement in response time afforded by the use of the neuroadaptive automation is given in Table 7. In trials in which there was a miss, neuroadaptive automation was not employed and the original response time was used. The mean response time difference was calculated from the original onset time minus the onset of the neuroadaptive automation for all perturbation piloting trials. The mean improvement in response time across participants was a reduction from 425 to 353 ms under neuroadaptive automation, or an average of 72 ms improvement. Figures 5A,B depicts the decoded response times plotted on the single trial activation waveforms of the adaptive automation (black circles) for participant 1 (best performer) and 3 (median performer), respectively. The single trials are arranged in increasing order of behavioral response time from bottom to top (white line). The significance of the time savings was evaluated by comparing the neuroadaptive automation response time difference (to that of the control stick response) relative to the distribution of response time differences of over 1000 models trained with randomly permuted labels (See Methods Section). The p value was computed by the number of times the models with permuted labels had larger response time differences than the BCI trained with the correct labeling over the 1000 permuted models (see Table 7). Table 8 shows the time savings of the six participants tested using the weights from the model of the best participant on the complex piloting task. The same permutation technique as discussed above was used to evaluate statistical significance. While all participants showed a significant difference in time savings even using a model trained by a different participant, the time savings were significantly (*p* < 0.05; paired Wilcoxon signed rank test) greater when using their own model (median = 62.4 ms; mean = 71.9 ms) vs. the generalized model (median = 35.0 ms; mean = 51.5 ms).

**Table 7:** **Improvement in response time by adaptive automation complex piloting task through the Grand Canyon**.

**ID**	**N**	**TP**	**FP**	**Org Mean (ms)**	**Org Se (ms)**	**BCI mean (ms)**	**BCI Se (ms)**	**BCI rt diff mean (ms)**	**BCI rt diff se (ms)**	**Perm rt diff Mean (ms)**	**Perm P**
1	60	47	1	357.9	5.2	283.1	9.8	74.8	7.8	5.2	0.002
2	52	22	2	436.5	13.8	369.7	15.6	66.7	14.3	6.4	0.023
3	58	38	4	454.0	10.2	405.2	13.6	48.7	7.9	4.5	0.014
4	58	46	7	359.8	7.3	277.0	10.6	82.8	10.4	4.9	0.001
5	59	32	6	462.2	6.6	404.2	12.7	58.0	11.8	3.1	0.003
6	55	45	6	480.4	7.3	341.4	14.1	138.9	15.4	13.5	0.002
7	56	24	4	424.1	9.3	388.0	13.3	36.1	8.6	7.4	0.041
Group mean	59	36	4	425.0	8.5	352.7	12.8	72.3	10.9	6.4	0.016

*The p-value in the last column denotes the significance of the time savings improvement of the BCI adaptive automation over the original joystick based response times based on permutation testing of 1000 models trained with random labels*.

*ID, Participant identification number; N, Number of Perturbation Piloting Trials; TP, True Positives (hits); FP, False Positives (false alarms); rt diff, response time difference; Org, Original; se, standard error; Perm, Permuted; BCI Brain Computer Interface*.

*The Perm P-value for the group mean is the paired Wilcoxon signed rank test comparing the BCI rt diff values to the Perm rt diff values for the seven participants*.

**Table 8:** **Generalization of performance using best subjects weights: improvement in response time by adaptive automation complex piloting task through the Grand Canyon**.

**ID**	**N**	**TP**	**FP**	**Org Mean (ms)**	**Org Se (ms)**	**BCI mean (ms)**	**BCI Se (ms)**	**BCI rt diff mean (ms)**	**BCI rt diff se (ms)**	**Perm rt diff Mean (ms)**	**Perm P**
1	–	–	–	–	–	–	–	–	–	–	–
2	52	9	0	436.5	13.8	408.7	15.6	27.8	10.7	7.9	0.026
3	58	35	3	454.0	10.2	407.3	13.5	46.7	7.7	5.5	0.009
4	58	27	2	359.8	7.3	327.9	9.3	31.9	7.9	4.1	0.001
5	59	23	3	462.2	6.6	427.7	11.2	34.5	9.5	2.9	0.001
6	55	43	4	480.4	7.3	347.9	15.8	132.5	15.7	17.8	0.002
7	56	21	2	424.1	9.3	388.7	11.8	35.4	9.2	2.8	0.006
Group mean	56	26^*^ (35)	2^*^ (5)	436.2	9.1	384.7^*^ (364.3)	12.9 (13.3)	51.5^*^ (71.9)	10.1 (11.4)	6.8 (6.3)	0.032 (0.032)

*The p-value in the last column denotes the significance of the time savings improvement of the BCI adaptive automation over the original joystick based response times based on permutation testing of 1000 models trained with random labels*.

*ID, Participant identification number; N, Number of Perturbation Piloting Trials; TP, True Positives (hits); FP, False Positives (false alarms); rt, response time; Org, Original; se, standard error; Perm, Permuted; BCI Brain Computer Interface*.

*The Perm P-value for the group mean is the paired Wilcoxon signed rank test comparing the BCI rt diff values to the Perm rt diff values for the six subjects*.

*The number in parentheses are the group mean values of the original decoder excluding sub01*.

* *Denotes p < 0.05 on paired Wilcoxon signed rank test for the comparison between the original decoder and the one trained with sub01 model over the six participants*.

## Discussion

The present study examined whether it is possible to decode neural signals associated with the intention to act in response to an impending hazard. Using MEG, the results showed that neural activity could be decoded so as to decrease the time needed to respond to the hazard, compared to manual action. As such, the results demonstrate that neuroadaptive automation can be implemented to speed up intentional action when there is very little available to respond.

There has been extensive prior research showing the effectiveness of both neuroadaptive automation (Byrne and Parasuraman, 1996; Wilson and Russell, 2007; Ting et al., 2010) and passive BCI (Blankertz et al., 2010; Zander and Kothe, 2011) in enhancing human performance. However, the present study represents the first successful attempt to show that decoded neural activity can be used to potentially speed up split-second decision making in response to an impending hazard on a novel complex task that neither the participant or the classification model has been trained on. While the brain is indeed faster than the hand in responding to a hazard, its activity must be accurately decoded so as to accrue a savings in time. In the piloting task used in the present study, the mean savings in response time was 72 ms (ranging from 36.1 to 138.9 ms). Although this may seem relatively small, in situations where humans are moving at high speed toward a hazard, as in driving or piloting, the savings may be sufficient to avert disaster.

To put a time savings of 72 ms in context, consider the flight characteristics of a F22 aircraft on the complex piloting task. Table 9 gives the response times to the in-flight perturbation for each participant [Figures 5A,B depicts the decoded response times plotted on the single trial activation waveforms of the adaptive automation (black circles) for participant 1 (best performer) and 3 (median performer), respectively]. Even with an average improvement of 72 ms in response time this can result in an average savings of up to 7.4 m of lost altitude as a result of earlier initiation of recovery in attitude to the perturbation. This could make a difference between a successful and failed attempt to avoid a collision. It should be noted that the large variability in savings between participants is likely a result of the quality of the MEG data in terms of separating task related activity from artifacts rather than expertise on the task. There was no apparent relationship between the savings afforded by the simulated neuroadaptive automation and manual response time on the task. It is known that there is considerable variability in the quality of MEG and EEG data across individuals that impacts successful BCI performance (Lotte et al., 2013).

**Table 9:** **Flight characteristics of F22 on Grand Canyon task**.

**ID**	**Avg altitude at time of perturbation (m)**	**Avg airspeed at time of perturbation (Km/h)**	**Avg airspeed at time of perturbation (m/s)**	**Descent rate DR mean (m/s)**	**Descent rate DR greatest (m/s)**	**Avg response Improvement by BCI over all trials (ms)**	**BCI Savings in Descent Altitude based on Mean DR (m)**	**BCI Savings in Descent Altitude based on Greatest DR (m)**
1	81.4	1122	311.6	69.1	87.4	75	5.2	6.5
2	79.8	1082	300.7	65.4	94.7	67	4.4	6.3
3	80.2	1117	310.3	56.2	83.7	49	2.8	4.1
4	79.8	1115	309.8	79.7	103.2	83	6.6	8.6
5	82.4	1117	310.2	71.8	124.6	58	4.2	7.2
6	82	1122	311.7	75.4	111	139	10.5	15.4
7	79.9	1113	309.1	69.6	93.3	36	2.5	3.4
Group mean	80.8	1113	309.1	69.6	99.7	72	5.2	7.4

*ID, Participant identification number; DR, Decent Rate; Avg, average*.

*The climb/descent rate is variable depending on the attitude of the plane at time of perturbation. The values given are the (1) mean of the maximum slope of descent calculated over a 200 ms period across trials and (2) the greatest maximum slope of descent calculated over a 200 ms period across trials*.

*It is important to note that time and distance to ground saved by earlier elevator engagement is not only the savings in less descent toward ground but also allows for gain in altitude relative to time because of earlier climb*.

It must be acknowledged, however, that the improvement in response time using neuroadaptive automation comes at the expense of making false alarms on a small number of trials. As in any automated alarm system, the tradeoff between correct early warning (hits) and false alarms has to be considered when setting the decision criterion of the alarm (Swets, 1973; Parasuraman and Riley, 1997). It may be possible in some cases to adjust the criteria of the BCI-decoder to make less false alarms at the expense of making less hits as well and reducing the overall response time improvement afforded by the neuroadaptive automation. For example in the study by Blankertz et al. (2002) the classifier was trained such that it was optimal under the constraint that the false positive (false alarm) rate attains a preset value.

The presence of a false alarm by the BCI-decoder could be somewhat problematic. Without some type of system that would identify externally induced perturbations from changes in attitude induced by the pilot in flight the neuroadaptive automation would initiate a recovery maneuver. Which in this case is to reverse the pitch down elevator deflection caused by the perturbation. Without the presence of a real perturbation, if the plane was in level flight and the BCI decoder made a false-alarm the neuroadaptive automation would cause the plane to make an abrupt climb. With respect to the pilot, this would constitute a pitch up perturbation. The goal of the hypothesized neuroadaptive automation is not to take control away from the pilot but rather to speed up the response of the pilot's motor intentions to unexpected flight conditions such as perturbation of attitude. While the use of detecting error-related potentials to decrease error rate has been successful in some implementations (Blankertz et al., 2002; Parra et al., 2003) it, unfortunately, is not likely to be of benefit in detecting motor intention to improve response time. This is because the relevant features for detecting the error-related potential on a single trial basis is after the response is made. One way to possibly keep the pilot in the loop and reduce the effects of false-alarms is to engage the neuroadaptive automation for only a couple hundred milliseconds and immediately disengage it in response to opposite deflection of the flight controls by the pilot. This would reduce the detrimental effects of false-alarms and at the same time would speed up response to recover from true perturbations in the case of hits. Given that the BCI-decoder is extracting motor intention related activity it would be interesting to determine whether the pilot actually notices the engagement of the neuroadaptive automation in the case of hits or rather just feels that they are really fast in reacting. The extent to which pilot-automation induced oscillations arise and offset the beneficial affects of time savings of the neuroadaptive automation need to be investigated using closed-loop implementation of the system during flight simulation (It should be noted that our study reported here only uses an open-loop BCI decoder tested offline to test the feasibility of implementation in neuroadaptive automation).

Although the BCI-decoder was trained using a specified window (120 ms) centered at the time of the peak evoked response prior to movement onset to detect a perturbation causing a pitch down attitude while in straight and level flight over the ocean (simple visual field) it was able to generalize to a novel complex flight condition in which the pilot maneuvered the plane through the Grand Canyon. In this complex condition the orientation of the perturbation with respect to the horizon is dependent on the roll angle (bank angle) of the plane at the time of the perturbation. The magnitude of the perturbation reflected in negative deflection in the pitch axis is dependent on the planes attitude (pitch, roll, yaw axes), speed, airflow over the flight surfaces, and the time in which it takes for the pilot to initiate recovery (the longer it takes the bigger the perturbation effect). It is impressive the BCI-decoder is able to generalize to the novel complex flight condition given that the nature of the perturbation and the corresponding visual aspects of the scene and ongoing motor control are quite different from the training situation. As we envision the closed-loop operational neuroadaptive automation system it would not need to know the magnitude of the perturbation (although this information may be available by flight instruments) as its job is to only initiate recovery based on the decoded motor intention of the pilot. It is up to the pilot to appropriately control the plane within the first couple hundred milliseconds after the neuroadaptive automation has been initiated. As it stands now the system is only set up to recover from a pitch down attitude. Ideally, we would like a system that could recover from a perturbation in attitude to any of the axes (pitch, roll, yaw) or combinations thereof. By comparing data from flight instruments that precisely measure attitude of all axes of the plane and pilot directed control movements the neuroadaptive automation could initiate the proper combination of control surface deflections to recover from various types of non-pilot induced perturbations. It would be interesting to test whether our system would generalize to other types of perturbation in attitude even though it was only trained on a pitch down perturbation. While this system using constraints determined by flight instruments may work in the case of perturbations it may not be effective in situations involving collision avoidance (e.g., with another aircraft or bird, etc.). In these situations it would be necessary to additionally build a BCI-decoder that can determine the desired direction of motor intention as it relates to the flight controls governing the attitude of the plane. This task may be difficult to accomplish within the framework of achieving the desired time savings to initiate recovery as fast as possible.

For the complex flying task no information was given concerning the timing of the peak of the event related evoked response to the onset of the perturbation used during training. Rather, the 120 ms window of the BCI-decoder progressed through the data in 8 ms steps until it identified an occurrence of a perturbation. The initial time window for the perturbation present trials started at the onset of the perturbation (the onset of the perturbation absent trials was randomly determined). However, there is no implicit information in this starting time that would reference the time of the evoked response upon which the BCI-decoder was trained. The presence of false alarms for the perturbation absent trials may be problematic for application of neuroadaptive automation working in a continuous manner given that the occurrence of true perturbations is quite sparse. As was discussed above, one way to reduce the number of false alarms made by the BCI-decoder is to only attempt to decode motor intention at points in which a perturbation is detected by flight instruments and then the appropriate recovery maneuver is applied by the neuroadaptive automation. One could implement a system that automatically recovers from a perturbation without regards to the pilot's intention (“Automation”). However, this is not the intention of the neuroadaptive automation proposed here for which the goal is to always keep the pilot intentions in control of the aircraft.

Previous research conducted on detection of driver braking intention, using EEG (Haufe et al., 2011, 2014; Kim et al., 2015), is relevant to the discussion of our results. In their studies as well as in ours simple amplitude based features related to the onset of the visual event were used for decoding the onset of movement intention. The visual event signaling the onset to move in the Haufe et al. (2011, 2014) and Kim et al. (2015) studies was the flashing of the brake light on the car just in front of the one the participant was driving. In our study the visual event signaling the onset to move was the changes in the optic flow field and the change in the position of the horizon (sky relative to ground) (See Figure 2). The finding that the best participant's decoding model generalizes to the remaining six subjects on detecting perturbation on the complex flying task with significant, although reduced, time savings (See Tables 6, 8), does suggest that the features selected by the model are not individual specific but are to some degree common across participants. As it stands now at least one session of the simple flying task is necessary to extract the task related independent component that help in artifact extraction. However, the finding that the BCI-decoder generalizes across participants (See Tables 6, 8) is promising in future attempts to make a generic system that does not require training.

There are three important aspects that distinguish our study from that of previous studies investigating braking intention.

The first is that our test condition was on a novel task that was fairly different from the one the BCI-decoder was trained on rather than just using a subset of trials on the same task for testing as is commonly done in decoding studies (Garrett et al., 2003; Wilson and Russell, 2007; Haufe et al., 2011, 2014; Baldwin and Penaranda, 2012; Callan et al., 2015; Kim et al., 2015). Our study demonstrates that a BCI-decoder trained on a simple task can generalize to a more complex one characteristic of real world conditions with significant performance in identifying perturbation events (mean bacc = 73%, *p* < 0.05; mean d′ = 1.52; mean a′ = 0.84; Table 5) with a significant time savings of 72 ms (Table 7).

The second is that the testing session (complex piloting task) requires that the participant use the same control stick to recover from the perturbation as used to maneuver the plane tracking above the river. Under these conditions it is necessary for the BCI-decoder to be able to distinguish brain activity related to the perturbation and the intention to move from ongoing changes in the visual field and motor intention required to pilot the plane. This is substantially different from decoding of movement intention of the foot from the accelerator to the brake in response to a flashing light. In order to extract neural activity related to movement intention in response to a perturbation, rather than that just related to the visual event, the BCI-decoder was trained to distinguish between trials in which the participant was to pull back on the control stick in response to a perturbation vs. just passively viewing the perturbation. All but one of the subjects showed significant classification performance in identifying movement intention trials from passive viewing trials on the test session (mean bacc = 69.8%, *p* < 0.05; mean d′ = 1.30; mean a′ = 0.80; See Table 4). The ability of the BCI-decoder to be able to identify cases of motor intention in response to identical visual events likely contributes to its ability to distinguish between variations in brain activity in response to changes in the optic flow pattern and movement intention in response to a perturbation rather than changes in the optic flow pattern induced by piloting while maneuvering through the Grand Canyon.

The third is the difference in response time for emergency braking, that is approximately 650 ms (Haufe et al., 2011, 2014; Kim et al., 2015), compared to pulling back on the stick to recover from a perturbation, which took approximately 437 ms for the complex flying task and 369 ms for the simple flying task. One reason why the time savings in the braking studies [up to 222 ms using combined EEG and EMG (Haufe et al., 2014)] is larger than in our study (72 ms) may be attributed to the longer response time for emergency braking (over 200 ms longer). The mean peak of the event related potentials used as the target range to train the BCI-decoders in our study was 246 ms (See Figure 3B). Because of the relatively fast response times the slower event related potentials could not be used for decoding because they occur after the behavioral response has already been given. The mean response time for the complex flying task for the adaptive automation is 352.7 ms compared to the original of 425.0 ms. The mean time in which decoding performance reached an area under the curve (A′) value of 0.8 was also around 350 ms in the emergency braking studies (Haufe et al., 2011, 2014; Kim et al., 2015). It should be mentioned that the improvement in response time afforded by the adaptive automation in our study for some of the participants allowed them to have almost superhuman performance on this piloting task.

Given that the perturbation we employed abruptly alters the optic flow field we predict that visual motion processing areas as well as brain regions involved with motor intention (premotor cortex, motor cortex, somatosensory cortex, parietal cortex) are involved in decoding the decision for rapid movement in response to an impending hazard. While there is considerable variability in the extent and location of brain activity of the selected independent component used for the BCI-decoder for each participant there are regions that are commonly activated across the participants (See Figures 3, 4 and Tables 2, 3). Consistent with our predictions all subjects showed some degree of activity in visual motion processing areas (hOC5, MT, IOG), as well as the premotor cortex, pre-central gyrus (motor cortex), post-central gyrus (somatosensory cortex), and parietal cortex (superior parietal lobule) (See Figures 3, 4 and Tables 2, 3). The visual cortex (BA17,18) also showed some degree of activity from all subjects (See Figures 3, 4 and Tables 2, 3). Our findings are consistent with fMRI research investigating action intentions from preparatory brain activity (Gallivan et al., 2011). In the Gallivan et al. (2011) study, decoded activity from voxels in multiple parietal, premotor, and motor regions was found to successfully predict intended future grasp and reach movements. A study using electrocortiocography (ECoG) revealed that in addition to motor and premotor activity somatosensory activity also precedes voluntary movement (Sun et al., 2015). The finding of predominantly caudal rather than rostral dorsal premotor cortex activation found for most participants in our study (See Figures 3, 4 and Tables 2, 3) is interesting as it relates to studies showing that that action intention is processed more caudally and attention is processed more rostrally in the premotor cortex (Boussaoud, 2001).

A potential limitation of our study is the low number of participants. However, the primary aim of our study is to show the feasibility of the proposed approach for the development of neuroadaptive automation and to determine limitations that need to be addressed in future research. In our study the results from each individual participant are given. Even when individually tailored models were trained specifically on data from that participant there is some degree of variability in performance at predicting presence/absence of a perturbation (ranging from 63.7 to 85.6%, See Table 5), and the corresponding time savings (ranging from 36.1 to 138.9 ms, See Table 7), as well as the pattern of brain activation (See Figures 3, 4 and Tables 2, 3). In the future, it may be interesting to investigate why some participants have better predictive models than others. These results strongly suggest that best performance will be achieved by individually tailored systems rather than using a generalized system that works across individuals. The drawback of individually tailored systems is the time necessary to train the system including ICA and the BCI-decoder. While this study does demonstrate that it is potentially possible to enhance response time by using an off-line BCI-decoder in these select participants it will be necessary to test a larger sample to see how well they generalize to the population in general and to determine factors predicting model efficacy.

Given that the mean time savings is 72 ms in the simulated off-line open loop neuroadaptive automation system demonstrated here, it is important to discuss whether the processing time would be of sufficient speed to be used in a real-time closed loop neuroadaptive automation system (see Figure 1). The Yokogawa 400 channel MEG system at ATR is set up with a real-time processing system. The hardware and software for acquiring MEG channel data in real time and analog to digital conversion includes the following: National Instruments A/D Converter boards (6 Boards: 80 channels per board) can convert 400 MEG channels plus additional channels (EEG, EOG, triggers, etc.) sampled at 1000 Hz. To get high temporal precision that is stable the National Instruments real-time operating system “Pharlab” is used on a dedicated computer. Pharlab carries out filtering operations on 400 channel MEG and sends the analog to digital converted MEG channel data via UDP to a different computer for further processing in ~1.5 ms. The application of the ICA weight matrix of the selected independent component to the 400 channel MEG data as well as the weight matrix of the BCI-decoder over the computed activation waveform is < 0.1 ms. The ICA and BCI-decoder can operate in such a short time because the weights have been trained ahead of time based on data from previous sessions. Therefore, the data acquisition, preprocessing, and BCI-decoding can all be accomplished in < 2 ms in the real-time system. The X-Plane flight simulator is running at around 400 Hz. It takes ~2.5–5 ms for the flight simulator computer to receive the command from the BCI-decoder computer over UDP and initiate the directed command. Based on the specifications of the system at ATR the loss in time savings afforded by the simulated neuroadpative automation resulting from processing time would be ~4.5–7 ms. This would still leave a mean time savings afforded by the neuroadaptive automation ranging from 65 to 67.5 ms, which could be of substantial benefit in hazardous time critical situations. Most of the delay (resulting in reduction of time savings) is in the processing speed of the flight simulator, which theoretically could be improved if using dedicated hardware and software in a real aircraft.

While this system is specific to the MEG setup at ATR it is possible to make such a dedicated real-time system that will work with EEG that can be used in real-world settings. In order for the system to be feasible in real aircraft it will be necessary to use a more portable technology such as EEG. The signal processing techniques used in this study together with automatic subspace reconstruction have been shown to be able to separate artifacts from brain related activity in flight even in an open cockpit biplane (Callan et al., 2015). It is uncertain whether moving from 400 channels to 64 or 20 channels with an EEG setup will have a large effect on system performance. Source localization would likely be considerably worse in the case of EEG especially with 20 channels compared with that of MEG. The number of channels will also play an important part in the ICA in the number of brain and artifact components that can be separated. In future research we will test an EEG based closed-loop version of this neuroadaptive automation system on a motion platform based flight simulator to determine its feasibility and additional processing that may be necessary if it were to be realized in actual manned or unmanned aircraft.

## Conclusion

Our study explores the potential that neuroadaptive automation may have in facilitating human performance. Our goal is to develop a system that enhances performance to super human levels during normal hands on operation of an airplane (vehicle) by reducing the response time by directly extracting from the brain the movement intention in response to a hazardous event. This approach differs considerably from those that utilize BCI to maneuver a vehicle by hands-off control by such methods as decoded mental imagery or attention related steady state visual evoked potentials (Blankertz et al., 2010; LaFleur et al., 2013). These applications of BCI, although impressive, are severely limited in performance compared to normal hands on control with the addition of greater workload as well as divided attention away from the task at hand (It should be noted however, that these types of BCI are of extreme benefit when the normal channels of motor control are impaired). Advantages of the neuroadaptive automation BCI implementation proposed here, afforded by the use of only brain activity naturally occurring during the perceptual motor task, include improved performance with no additional workload or attentional demands for the pilot (operator), as well as no training by the pilot to fit the BCI. However, it should be noted that human training protocols for utilizing BCI are likely to improve performance (Lotte et al., 2013). Our proposed BCI-decoder works continuously over time without any a-priori knowledge of when a perturbation may occur. In addition it was shown to be able to generalize to more complex tasks and differentiate between motor intention to an unexpected perturbation from that used during normal maneuvering. Future research needs to test the proposed neuroadaptive automation online using EEG in motion based flight simulators as well as in real airplanes to evaluate its real-world performance. It is interesting to conjecture whether the participants will notice when the neuroadpative automation is active or will they just think they are responding really fast. This research adds to the growing field of neuroergonomics and specifically to aviation cerebral experimental sciences. Our results, using an off-line BCI-decoder, suggest that indeed neuroadaptive automation can be implemented that is faster than the hand. The data can be shared with interested scientists upon request.

## Author contributions

DEC, CT, and DBC designed and conducted experiment. DEC, CT, DBC, MS, and RP analyzed results of the experiment. DEC and RP wrote the manuscript.

## Funding statement

Funding for this research was supported in part by a contract with the National Institute of Information and Communications Technology, Japan, entitled, “Multimodal integration for brain imaging measurements” and by the Center for Information and Neural Networks, National Institute of Information and Communications Technology. MS was supported by the National Institute of Information and Communications Technology. Additional support to RP was given by the Air Force Office of Sponsored Research grant FA9550-10-1-0385.

### Conflict of interest statement

The authors declare that the research was conducted in the absence of any commercial or financial relationships that could be construed as a potential conflict of interest.
